# Alkylphenols (4-n-Nonylphenol and 4-n-Octylphenol) in Milk and Dairy Products, Beverages, and Vegetable Oils: Occurrence and Dietary Exposure in Türkiye

**DOI:** 10.3390/foods15061063

**Published:** 2026-03-18

**Authors:** Oltan Canlı, Barış Güzel, Burhan Basaran

**Affiliations:** 1 Climate Studies and Water Management Research Group, Climate and Life Vice Presidency, TUBITAK Marmara Research Center, 41470 Kocaeli, Türkiye; oltan.canli@tubitak.gov.tr (O.C.); guzelbaris08@gmail.com (B.G.); 2 Department of Nutrition and Dietetics, Faculty of Health Sciences, Recep Tayyip Erdogan University, 53100 Rize, Türkiye

**Keywords:** endocrine-disrupting compounds, environmental contaminants, human exposure assessment, packaging migration

## Abstract

Alkylphenols, including 4-n-nonylphenol (4-n-NP) and 4-n-octylphenol (4-n-OP), are endocrine-disrupting chemicals that can migrate from the environment and food contact materials into food, posing potential public health risks. A total of 158 food samples were analyzed concerning the levels of these two chemicals, including milk and dairy products (*n* = 54), beverages (*n* = 79), and vegetable oils (*n* = 25). Average 4-n-NP/4-n-OP concentrations followed the order: vegetable oils (0.28 ± 0.24/0.76 ± 0.82 µg/L) > beverages (0.17 ± 0.20/0.24 ± 1.32 µg/L) > milk and dairy products (0.13 ± 0.26/0.23 ± 0.47 µg/L). Olive oil and ready-to-drink (RTD) chilled coffee showed the highest contamination levels within their categories, while UHT milk (4-n-NP) and ayran (4-n-OP) were notable among dairy products. Plastic and metal can containers were associated with higher alkylphenol migration, particularly in oily foods and some beverages, whereas carton packaging generally showed lower levels. Dietary exposure assessment indicated that the combination of high consumption and high contamination (e.g., RTD chilled coffee, energy drinks, ayran) markedly increased exposure risk. This study provides the first comprehensive comparative assessment of 4-n-NP and 4-n-OP contamination in multiple food categories in Türkiye, highlighting both product-specific and packaging-related risks.

## 1. Introduction

4-n-nonylphenol (4-n-NP) and 4-n-octylphenol (4-n-OP), which are among the alkylphenol derivatives, have a wide range of industrial uses, primarily in detergent production, industrial cleaners, emulsifiers, pesticide formulations, and plastic additives, due to their high surface-active properties and chemical stability [1]. These compounds are widely found in the environment as degradation products of nonionic surfactants [2]. Due to their low water solubility, pronounced lipophilic nature and biopersistent properties, they have a high potential for bioaccumulation and biomagnification [3,4,5]. These properties may lead to long-term toxicological risks to human health via environmental exposure or through the food chain.

4-n-NP and 4-n-OP represent the linear (normal-chain) isomers of 4-nonylphenol (4-NP) and 4-octylphenol (4-OP), respectively. 4-NP and 4-OP are endocrine disruptors that act through estrogen receptors and are associated with reproductive system disorders, metabolic dysfunctions, and potential tumorigenic effects [6,7,8]. Recent in vitro and cellular studies have shown that 4-OP, in particular, triggers endoplasmic reticulum (ER) stress, disruptions in calcium homeostasis, and apoptosis in human cells, while 4-NP can affect cellular proliferation and neoplastic transformation processes [9,10]. Epidemiological reviews indicate a possible relationship between 4-NP exposure, obesity, and certain types of cancer [11]. All considering, these findings indicate that under low-dose but chronic exposure conditions, 4-NP/4-OP may pose a considerable risk to public health through food and environmental pathways, warranting a cautious approach.

The main pathways for 4-n-NP and 4-n-OP to enter the food chain are environmental contamination (via water, soil, and air), food-contact surfaces during processing, packaging materials, and water used in the production process [12,13,14]. Recent studies have revealed that 4-n-NP and 4-n-OP residues are detected at varying levels in food and beverages in both developed and developing countries [15,16,17,18].

Milk and dairy products are of particular importance as they are essential sources of protein and fat in the human diet. 4-n-NP and 4-n-OP residues detected in these products pose a public health risk due to the endocrine-disrupting and toxicological effects of long-term exposure. Similarly, beverages and vegetable oils play a critical role in nutrition by meeting the body’s energy needs, maintaining fluid balance, and providing essential nutrients [19,20]. Chemical contamination in these foods can lead to metabolic disorders, hormonal imbalances, and other long-term health problems in the event of chronic exposure. Furthermore, it should be remembered that dietary exposure is a lifelong process. Since infants, children, and pregnant individuals are particularly susceptible to contaminants, determining foodborne exposure is crucial not only for toxicological assessments but also for shaping public health policies [21,22]. In this context, the presence of 4-n-NP and 4-n-OP in food is not only a matter of analysis but also a public health concern requiring the development of strategies for sustainable agriculture, safe food production, and consumer health protection. Therefore, regular monitoring of these contaminants in commonly consumed foods such as milk and dairy products, beverages, and vegetable oils, along with exposure assessments and the establishment of risk management policies, is of great importance [16,23,24].

The aim of this study is to determine the levels of 4-n-NP and 4-n-OP in milk and dairy products, beverages, and vegetable oils from different brands sold in Türkiye and to calculate the doses to which the population is exposed through the consumption of these foods. This study aims not only to scientifically document the current situation but also to provide data for food safety and public health policies. It is anticipated that the data obtained will form the scientific basis for measures to be taken (e.g., improving production processes, setting maximum residue limits) and will significantly contribute to the protection of public health. Therefore, in this study, foods that have an important place in the human diet and are prone to contaminant accumulation due to their lipophilic structure were examined, with particular focus on milk and dairy products, vegetable oils, and beverages that carry a high risk of exposure due to raw materials, packaging, and processing. In this study, the analytical scope was restricted to the linear isomers 4-n-NP and 4-n-OP, which can be reliably quantified using available reference standards and a validated GC–MS/MS method.

## 2. Materials and Methods

### 2.1. Materials

In this study, 158 products were analyzed and selected based on their availability and brand recognition in Türkiye. All samples were purchased from nationwide chain supermarkets operating across Türkiye, with two units collected for each product in their original, unopened packaging. Sampling was conducted between August and November 2025, with products purchased in groups over this period. All samples were stored in their original packaging under product-specific storage conditions and analyzed before the end of their shelf life. Milk and dairy product samples (*n* = 54) included UHT milk (*n* = 15), children’s flavoured milk (*n* = 17), yogurt (*n* = 12), kefir (*n* = 6), and buttermilk (ayran) (*n* = 4). Beverage samples (*n* = 79) consisted of soft drinks (*n* = 28), fruit juice (*n* = 19), energy drinks (*n* = 9), bottled water (*n* = 8), ready-to-drink (RTD) chilled coffee (*n* = 8), and iced tea (*n* = 7). Vegetable oil samples (*n* = 25) included sunflower oil (*n* = 9), corn oil (*n* = 7), and olive oil (*n* = 9). Dairy product samples 1–22 were sold in plastic packages, while samples 23–54 were sold in carton packages (Tetra Pak); beverage samples 55–65, 79, 80, 94, 103–110, and 126–133 were sold in plastic packages; samples 66–78, 95–102, and 111–125 were sold in metal can packages; samples 81–93 were sold in carton packages (Tetra Pak); and vegetable oil samples 134–158 were sold in plastic packages. More information about the products, including packaging volumes, is shown in Tables S1–S3.

### 2.2. Analysis

#### 2.2.1. Reagents and Chemicals

All chemicals and solvents used in the analyses were of high purity, comprising either analytical grade or gas chromatography–mass spectrometry (GC-MS) grade. 4-n-NP (≥99% purity) and 4-n-OP (≥97% purity) standards were purchased from Sigma-Aldrich (St. Louis, MO, USA). The internal standard (IS), phenol-d6 (≥98% atom D), was obtained from Dr. Ehrenstorfer GmbH (Augsburg, Germany). Stock standard solutions (1 µg/mL) were prepared in high-purity GC-MS-grade methanol and stored at −20 °C in amber glass vials under dark conditions. Working solutions were freshly prepared by appropriate dilution of stock standards in methanol prior to analysis. GC-MS-grade methanol, acetonitrile, and n-hexane were purchased from Merck (Darmstadt, Germany). Anhydrous sodium sulfate used for drying organic extracts was pre-cleaned by heating at 400 °C for 4 h and stored in a desiccator. No derivatization was required for GC-MS/MS analysis. All glassware used during sample preparation and extraction was thoroughly rinsed with methanol and baked at 300 °C to eliminate potential contamination by phenolic compounds.

Calibration standards were prepared in matrix-matched solutions appropriate to each sample type. Procedural blanks and quality control samples were analyzed alongside the relevant samples to ensure the accuracy and precision of the measurements.

#### 2.2.2. Analytical Instrumentation

Quantitative and qualitative analysis of 4-n-NP, 4-n-OP, and phenol-d6 was performed using a Thermo Scientific™ TRACE™ 1310 gas (Thermo Fisher Scientific, Waltham, MA, USA) chromatograph coupled to a TSQ™ 8000 triple quadrupole mass spectrometer (Thermo Fisher Scientific, Waltham, MA, USA) equipped with an electron ionization (EI) source and operated in multiple reaction monitoring (MRM) mode. Chromatographic separation was achieved using a TG-5MS column (30 m × 0.25 mm i.d., 0.25 µm film thickness; Thermo Fisher Scientific, Waltham, MA, USA). The oven temperature program was as follows: initial temperature 60 °C (held for 1 min), ramped at 10 °C/min to 280 °C, and held for 10 min. The total run time was 25 min. Helium (≥99.999%) was used as the carrier gas at a constant flow rate of 1.0 mL/min. The injector was operated in splitless mode at 250 °C. The transfer line and ion source temperatures were maintained at 280 °C and 250 °C, respectively. Electron ionization was performed at 70 eV. The collision gas was high-purity argon.

**Figure 1:**
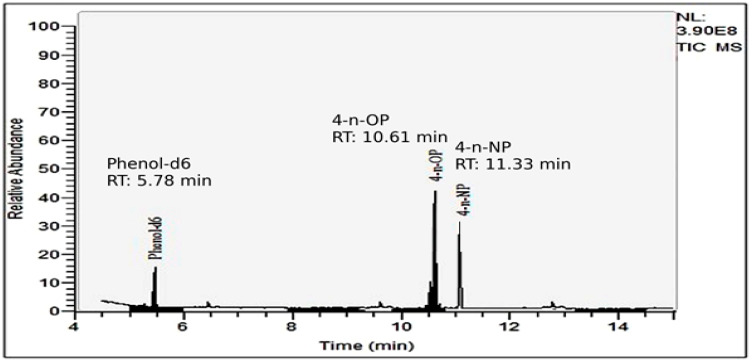
GC–MS total ion chromatogram of a soft drink (orange-flavored) sample (Sample 71) showing the detection of 4-n-NP (0.14 µg/kg) and 4-n-OP (0.40 µg/kg) under optimized analytical conditions.

Detailed tandem mass spectrometry parameters for each compound are provided in Table 1. The identification and quantification of 4-n-OP, 4-n-NP, and phenol-d6 were performed by GC-MS/MS in multiple reaction monitoring (MRM) mode using electron ionization (EI, 70 eV). The optimized retention times were 10.61 min for 4-n-OP, 11.33 min for 4-n-NP, and 5.78 min for phenol-d6. All compounds were analyzed in positive ion mode. Quantification was based on the most intense product ion (quantifier), with an additional qualifier ion used for confirmation. Specifically, MRM transitions were 206 → 121 (quantifier) and 206 → 135 (qualifier) for 4-n-OP, 219 → 133 and 219 → 147 for 4-n-NP, and 100 → 84 and 100 → 66 for phenol-d6. A dwell time of 50 ms and a collision energy of 10 eV were applied for each transition. These parameters provided high selectivity and sensitivity for the determination of target analytes in complex food matrices (Figure 1).

It should be noted that the chromatographic method applied in this study was op-timized for the determination of the linear isomers (4-n-NP and 4-n-OP) using corre-sponding analytical standards. Due to the structural complexity of technical nonylphenol mixtures, which contain multiple branched positional isomers, complete chromato-graphic separation of all NP isomers was not targeted under the applied GC conditions. The multiple peaks observed in the 4-NP chromatogram are consistent with the presence of different isomeric forms. However, quantification was performed exclusively based on the retention time and compound-specific MRM transitions of the linear 4-n-NP standard. Therefore, the reported concentrations represent linear 4-n-NP rather than total nonylphenol.

#### 2.2.3. Sample Preparation and Analysis

Sample preparation was optimized for the determination of 4-n-OP and 4-n-NP using GC-MS/MS with high recovery and minimal matrix interference. All samples were homogenized prior to extraction.

For dairy products (including milk, yogurt, kefir, etc.), a 10 g aliquot of the homogenized sample was placed in a 50 mL polypropylene centrifuge tube. Following this, 10 mL of GC-MS-grade methanol and 50 µL of phenol-d6 internal standard (1 µg/mL) were added. The mixture was vortexed for 2 min and subjected to ultrasonication for 15 min, after which it was centrifuged at 4000 rpm for 10 min at 4 °C. The supernatant was transferred and extracted twice with 5 mL of n-hexane. The hexane layers were combined, dried over anhydrous sodium sulfate, evaporated to 1 mL under a gentle nitrogen flow, and then transferred into amber glass vials for GC-MS/MS analysis.

For beverages (including soft drinks, juices, RTD chilled coffee, etc.), a 10 mL sample was blended with 10 mL of methanol and 50 µL of the internal standard solution. After vortexing for 1 min, liquid–liquid extraction was performed using 5 mL of n-hexane (two times). The organic layers were consolidated, dried with sodium sulfate, and evaporated under nitrogen to reach a final volume of 1 mL in n-hexane.

For vegetable oils (sunflower, corn, olive), about 2 g of oil was combined with 10 mL of methanol and 50 µL of an internal standard. Following vigorous shaking and centrifugation, the methanolic phase underwent solid-phase extraction (SPE) utilizing preconditioned C18 cartridges (500 mg). The eluted fraction was extracted with 5 mL of n-hexane, dried using sodium sulfate, and concentrated to 1 mL under a nitrogen atmosphere (Merck, Darmstadt, Germany). The final extract was preserved in n-hexane within amber vials.

All sample extracts were stored in amber glass vials at 4 °C and analyzed within 24 h to prevent degradation of target compounds. Each sample was independently extracted and analyzed in duplicate to assess method precision and ensure data reliability. Reported concentrations represent the mean values of two independent determinations.

#### 2.2.4. Quality Assurance/Quality Control (QA/QC)

Strict quality assurance and quality control (QA/QC) procedures were implemented throughout the analytical process to ensure data reliability and reproducibility across different matrices. To prevent contamination from labware, all glassware used during sample preparation was pre-cleaned by rinsing with methanol and baked at 300 °C. Extracts were stored in amber vials at 4 °C and analyzed within 24 h to minimize degradation of analytes. Calibration curves matched to the matrix were created by spiking blank samples of each matrix type (dairy, beverage, and oil) with seven concentration levels, which ranged from 1 to 500 µg/kg. Blank matrices were first analyzed to confirm that target analytes were not detected or were present at levels below the LOQ prior to their use for calibration. Matrix-matched calibration standards were prepared by fortifying these verified blank samples with appropriate volumes of mixed standard solutions before extraction, and the spiked samples were subjected to the entire extraction and analytical procedure. The calibration curves revealed excellent linearity, with correlation coefficients (R^2^) greater than 0.995 for all analytes (4-n-NP: 0.9964; 4-n-OP: 0.9971). Quantification was performed through IS calibration using phenol-d6. Each analytical batch consisted of 5–6 samples and included one procedural blank and one complete matrix-matched calibration series. In addition, at least one spiked QC sample per matrix type was analyzed within each batch to monitor ongoing method performance. All samples were independently extracted and analyzed in duplicate, and instrumental injections were performed in duplicate to verify repeatability. Procedural blanks were included with every batch of 5–6 samples to monitor potential contamination during sample preparation and instrumental analysis. No target compounds were detected in the blanks. Recovery studies were executed by spiking representative blank samples from each matrix category (n = 3 per matrix) at a concentration level of 50 µg/kg. The mean recoveries were found to be between 82% and 106%, with relative standard deviations (RSDs) below 10%, signifying good accuracy and precision. Instrumental limits of detection (LOD) and limits of quantification (LOQ) were calculated using signal-to-noise ratios (S/N = 3 for LOD, S/N = 10 for LOQ). LODs ranged from 0.01 to 0.10 µg/kg, and LOQs from 0.03 to 0.35 µg/kg, depending on the compound and matrix. In calculating the mean 4-n-NP and 4-n-OP levels of the samples, values below the LOQ were considered zero.

### 2.3. Dietary Estimated Daily Intake

Dietary exposure was calculated using the following Equation (1).

(1) $$\mathrm{EDI}=\frac{C\times \mathrm{IR}}{\mathrm{bw}}$$

C represents the average levels of 4-n-NP and 4-n-OP in each sample (µg/L), IR denotes the intake rate of each sample (mL/day), and bw refers to body weight (kg). The analyzed milk and dairy products and beverages are marketed as single-use items. Therefore, their consumption amounts were assumed to correspond to the portion sizes indicated on the packaging (Tables S1 and S2). Consumption data for vegetable oils were obtained from the Türkiye Nutrition and Health Survey [25] (Table S3). The average body weights of children (aged 3–6 years) and adults (aged 18 and over) were assumed to be 17.5 kg and 70 kg, respectively. In calculating dietary 4-n-NP and 4-n-OP exposure, values below LOQ in the samples were considered zero.

### 2.4. Data Analysis

The study data were analyzed using IBM SPSS Statistics version 26. Comparisons between independent groups were performed using the Mann–Whitney U test and the Kruskal–Wallis test. Post hoc pairwise comparisons were conducted with the Bonferroni test. Different letters or numbers within the same group indicate statistically significant differences (*p* < 0.05).

## 3. Results and Discussion

It should be noted that many studies were cited for comparative reporting of 4-NP and 4-OP without explicitly distinguishing between linear and branched isomers. However, closer examination of the analytical methodologies in several of these studies suggests that linear standards (4-n-NP and/or 4-n-OP) were likely employed, despite the use of generic terminology in the reported results. This inconsistency in nomenclature introduces uncertainty into direct comparisons and should be taken into account when interpreting differences across studies. Accordingly, comparisons in this study were performed in strict accordance with the terminology used in the reference studies. When 4-n-NP or 4-n-OP were explicitly reported, the data were compared directly; when only 4-NP or 4-OP were reported, the original nomenclature adopted in the respective studies was retained.

### 3.1. Milk and Dairy Products

**Figure 2:**
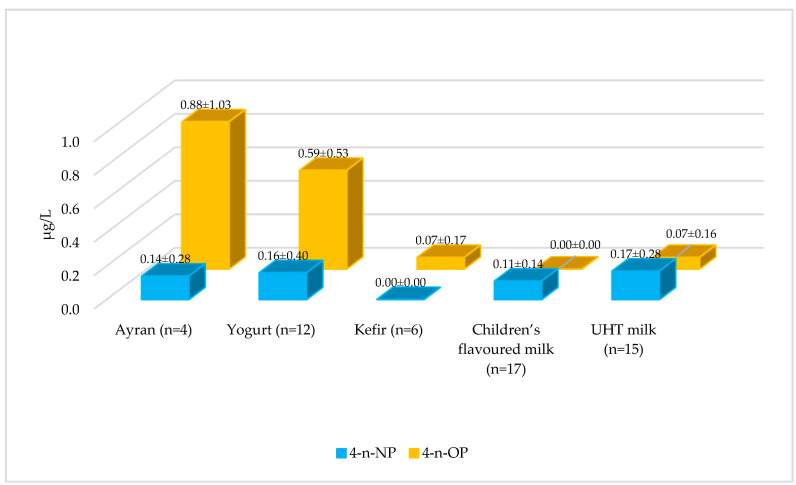
Average levels of 4-n-NP and 4-n-OP in milk and dairy products.

According to the data presented in Figure 2 and Table S4, 4-n-NP levels were determined to be <LOD in 74% of the milk and dairy products examined. According to the average 4-n-NP values, milk and dairy products were ranked as follows: UHT milk > yogurt > ayran > children’s flavoured milk > kefir. 4-n-NP levels in yogurt samples were significantly higher than in ayran, children’s flavoured milk, and kefir (*p* < 0.05). Compared to the literature, Casajuana and Lacorte (2004) reported higher average 4-n-NP levels in 5 milk samples (16.5–34.8 µg/kg) than in this study [26]. In a study conducted in Greece, a total of 27 dairy products, including milk, yogurt, children’s flavoured milk, and ice cream, were examined, and the average 4-NP level was reported as quite high (297 ± 130 µg/kg) [27]. These differences are likely due to regional contamination profiles. No studies reporting 4-n-NP and 4-NP levels in ayran and kefir could be found.

4-n-OP levels were determined to be <LOD in 67% of the milk and dairy products examined, which is a higher proportion than observed for 4-n-NP. The highest average 4-n-OP levels were found in ayran samples. These high values may be due to differences in the production or storage conditions of the samples. Statistical analyses showed that the 4-n-OP levels of ayran samples were significantly higher than those of other dairy products, and yogurt samples were significantly higher than kefir, children’s flavoured milk, and UHT milk (*p* < 0.05) (Figure 2, Table S4). These findings indicate that 4-n-OP accumulation may vary depending on product type and processing methods. Compared with the literature, 4-OP was not detected in sheep and buffalo milk in studies conducted in the UK and Italy [28,29]. In a study examining six types of milk and milk powder, 4-OP was detected in only two milk powder samples (1.5 and 0.3 μg/kg) and one milk sample (29.5 μg/kg) [30]. As an important gap in the literature, no studies reporting 4-n-OP levels in yogurt, ayran, kefir, and children’s flavoured milk samples were found. This makes the present study one of the pioneering studies documenting 4-n-OP contamination profiles in fermented dairy products.

The findings indicate that phenolic compound contamination in dairy products varies significantly depending on the product type, and each product group carries a unique risk profile. It is particularly noteworthy that 4-n-OP levels are higher in fermented products such as ayran and yogurt. While UHT milk exhibits higher 4-n-NP contamination levels, its overall contamination is lower than that of ayran and kefir. Kefir and infant milk, on the other hand, have lower average levels of 4-n-NP and 4-n-OP, and represent the safest product groups. It is believed that phenolic compound contamination can be reduced through optimization of production processes and appropriate packaging selection.

### 3.2. Beverages

**Figure 3:**
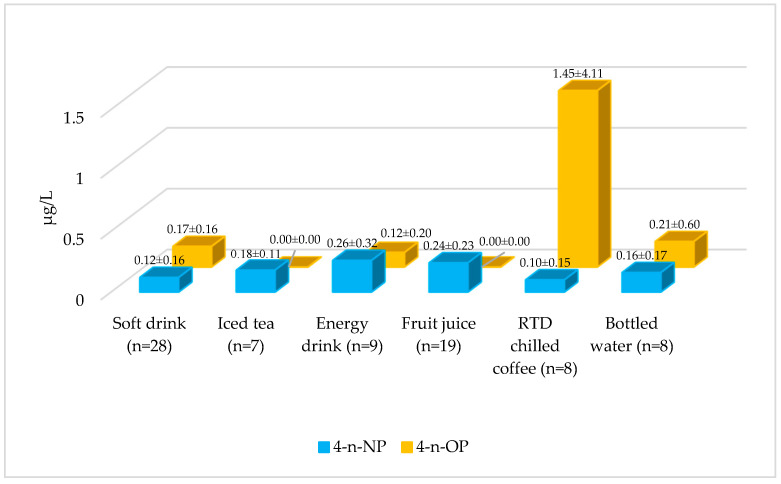
Average levels of 4-n-NP and 4-n-OP in beverages.

Of the beverages examined, 43% had 4-n-NP levels below the LOD, while the remaining 57% contained 4-n-NP. 4-n-NP levels in beverage samples ranged from <LOD to 0.71 µg/L. According to the average 4-n-NP level, beverages were ranked as follows: energy drinks > fruit juice > iced tea > bottled water > soft drinks > RTD chilled coffee (Figure 3, *p* < 0.05, Table S5). In a study conducted in France, no alkylphenols were detected in 25 bottled water samples [31]. The levels of 4-NP in different beverages were reported as 0.11–0.30 and 0.18–0.93 µg/L by Li et al. and Mohammadipour et al., respectively [17,32]. In another study conducted in Iran, 4-NP was detected in all 60 fruit juices examined. The average 4-NP level was reported as 1.42 ± 1.50 µg/L, which is considerably higher than the present study [14]. In a study conducted in China, 4-NP was detected in only two soft drink samples (detection rate = 33%), and the 4-NP levels in these two samples were reported as 0.02 and 0.006 µg/L [31]. In a study conducted in Germany, the average 4-NP level in brewed coffee samples was reported as 0.30 µg/L, while it was reported as 0.10–0.70 µg/L in different fruit juices [33]. In a study conducted in Australia, which examined 162 different beverages (soft drinks, energy drinks, bottled water, coconut water, sports drinks, and flavored water), alkylphenols were not detected [34]. Differences in these studies may be due to production processes, packaging materials, water source characteristics, and legal limits between countries.

4-n-OP levels were below the LOD in 67% of the examined beverages, while 4-n-OP was detected in the remaining 33%. The concentrations in beverage samples ranged from <LOD to 11.6 µg/L. The highest average 4-n-OP level was detected in RTD chilled coffee (Figure 3). Statistically, 4-n-OP levels in soft drink, RTD chilled coffee, and bottled water groups were significantly higher than those in the other beverage groups (*p* < 0.05; Table S5). A study conducted in the Czech Republic found no trace of 4-OP in bottled water samples [35]. The average 4-OP concentration in soft drinks observed here is consistent with the findings of Lv et al., who detected 4-OP in only one cola sample (0.20 µg/L) and one green tea sample (0.007 µg/L) among six soft drink products analyzed [31]. Variations between studies are likely attributable to differences in production processes, the chemical composition of water sources, packaging materials, and storage conditions.

The findings indicate that the distribution of 4-n-NP and 4-n-OP in beverages varies significantly by product type, with statistically significant differences observed. The most striking result is the very high 4-n-OP levels detected in the RTD chilled coffee group. The markedly higher 4-n-OP levels observed in the RTD chilled coffee group may be linked to matrix and formulation characteristics. RTD chilled coffee is typically an emulsified product containing milk/cream components and a fat phase. Differences in formulation (e.g., fat content and stabiliser/emulsifier use) may also influence the observed range. The energy drink group had the highest mean 4-n-NP. The elevated 4-n-OP levels in some bottled water samples suggest potential contributions from packaging migration or post-filling contamination. It is noteworthy that iced tea and fruit juice samples exhibited high 4-n-NP but low 4-n-OP levels. These product-specific differences are likely attributable to variations in water source, additive content, packaging type, and processing parameters.

### 3.3. Vegetable Oils

**Figure 4:**
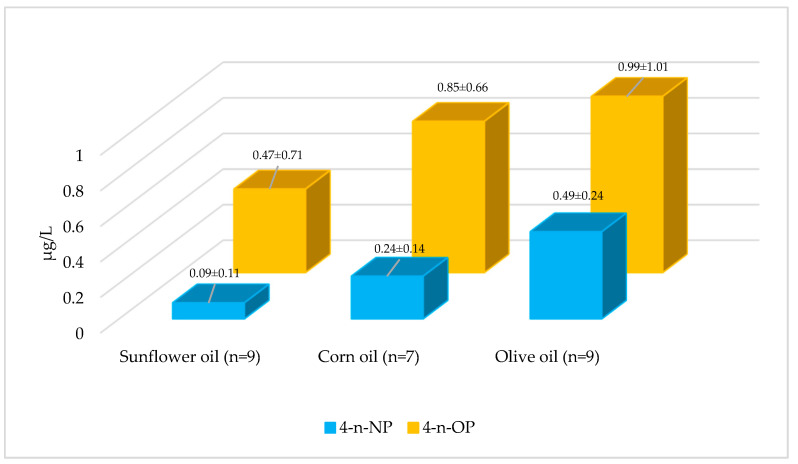
Average levels of 4-n-NP and 4-n-OP in vegetable oils.

4-n-NP levels were below the LOD in 24% of the examined vegetable oils, while 4-n-NP was detected in the remaining 76%. According to the average 4-n-NP level, vegetable oils were ranked as olive oil > corn oil > sunflower oil (Figure 4, *p* < 0.05, Table S6). In a study conducted in China, 4-NP was not detected in different vegetable oils [36]. In the Republic of Korea, the average 4-n-NP level in oils and fats (soybean oil, sesame oil, and butter) was 0.15 µg/kg (range: <LOD–1.14 µg/kg) [24]. Therefore, the 4-n-NP levels detected in vegetable oil samples in this study were lower than those reported in many previous studies. These differences may be due to variations in the geographic origin of the raw material, production and refining processes, storage conditions, and packaging materials.

4-n-OP levels were below the LOD in 24% of the examined vegetable oils, while 4-n-OP was detected in the remaining 76%. The concentrations varied between <LOD and 3.37 µg/kg. According to the average 4-n-OP level, vegetable oils were ranked as olive oil > corn oil > sunflower oil (Figure 4, *p* < 0.05, Table S6). In a study examining 20 vegetable oil samples, 4-OP was not detected [36]. Another study analyzing soybean oil, peanut oil, olive oil, and corn oil reported 4-OP levels ranging from <LOD to 2.10 µg/L [37]. Therefore, the 4-n-OP levels detected in vegetable oil samples in this study were higher than those reported in some previous studies. These differences may be attributed to variations in raw material origin, production and refining processes, packaging type, and storage conditions.

The three vegetable oil types examined in the study showed significant differences in 4-n-NP, and 4-n-OP levels. The highest contamination levels were detected in olive oil, while the lowest were found in sunflower oil. Corn oil and olive oil exhibited similar 4-n-OP levels, whereas sunflower oil showed lower contamination. The findings suggest that olive oil poses a higher risk for 4-n-NP and 4-n-OP contamination, while sunflower oil presents a relatively safer profile. The higher 4-n-NP and 4-n-OP levels observed in olive oil may be linked to processing and handling factors. Compared with other vegetable oils, olive oil is produced through a largely mechanical process (e.g., crushing and malaxation) with prolonged contact times and multiple handling steps, which may increase opportunities for contamination along the production line. In addition, olive oil is often stored for longer periods, and extended storage in plastic packaging may further influence the measured levels. These findings further suggest that industrial and environmental conditions during production and processing may play a decisive role in 4-n-NP and 4-n-OP contamination of vegetable oils.

In our dataset, 4-n-NP and 4-n-OP concentrations were higher in vegetable oils than in beverages and dairy products. This pattern is consistent with the lipophilic character of these compounds. Lipophilic chemicals preferentially partition into fat-rich matrices rather than water-based foods, so they tend to be detected at higher concentrations in oils. By contrast, beverages and most dairy products contain a larger aqueous fraction, which can limit the retention of these compounds and result in lower measured levels.

### 3.4. 4-n-NP and 4-n-OP Levels According to Packaging Types

**Figure 5:**
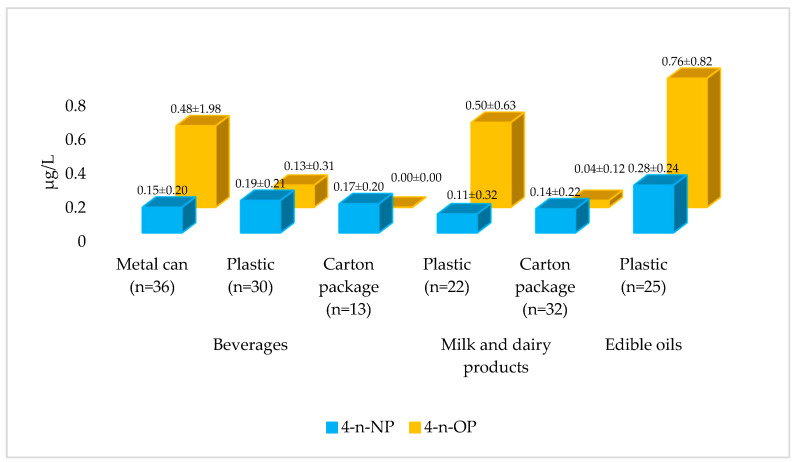
Average levels of 4-n-NP and 4-n-OP according to packaging types.

4-n-NP levels in plastic-packaged milk and dairy products were significantly higher than those in carton packaged products (*p* < 0.05) (Figure 5; Table S7). The literature has similarly reported higher 4-n-NP levels in milk samples stored in HDPE bottles compared to carton packaging [27]. Reported NP levels have ranged from 0.02 to 4.19 µg/kg across 11 different packaging types, including plastic and carton package [38]. Therefore, the information obtained for milk and dairy product packaging in this study is consistent with previous literature. However, some studies have also reported higher 4-NP levels in carton packaged dairy products than in plastic or metal can packaging, suggesting that differences may be related to interactions between packaging material properties and the food matrix [28]. Furthermore, higher 4-n-NP and 4-n-OP levels were detected in dairy products stored in plastic packaging compared to carton package, whereas alkylphenol levels were significantly lower in products stored in glass packaging [39].

According to the average 4-n-NP levels, beverage packaging was ranked as plastic > carton package > metal can, whereas milk and dairy product packaging was ranked as carton package > plastic (Figure 4). No statistically significant difference was observed between the 4-n-NP levels of packaging types in the beverage group (*p* > 0.05) (Figure 5, Table S7). Shabani et al. reported that plastic packaging for fruit juice contained higher levels of 4-NP than carton packaging, with this difference being statistically significant for some fruit juices [14]. Therefore, the general trend observed in this study regarding packaging types in the beverage group is consistent with the literature. However, the lack of a statistically significant difference may be attributed to the limited number of samples, differences in product content, or variations in the production parameters of the packaging materials.

When products in plastic packaging were compared, the highest average 4-n-NP level was observed in vegetable oils, followed by beverages and milk and dairy products (*p* < 0.05). For carton packaging, the lowest average 4-n-NP level was observed in milk and dairy products, while the highest was in beverages. However, no statistically significant difference was found between the groups (*p* > 0.05) (Figure 5, Table S7).

The lowest and highest average 4-n-OP levels in the beverage group were observed in carton package and metal can packages, respectively, while in the milk and dairy products group, the order was plastic > carton package (Figure 5). The absence of 4-n-OP in beverages packaged in carton package supports the protective properties of this packaging type. In the beverage group, 4-n-OP levels in metal can packages were higher than those in plastic and carton package, whereas in milk and dairy products, plastic packaging showed higher 4-n-OP levels than carton package (*p* < 0.05, Table S7). When comparing plastic packaging across different product groups, the lowest average 4-n-OP levels were detected in beverages, while the highest were found in vegetable oils (*p* < 0.05). In addition, 4-n-OP levels in milk and dairy products sold in carton packaging were higher than those in beverages (*p* < 0.05, Table S7). These findings may be explained by the reactivity of metal can packaging with acidic products (e.g., iced coffee), the higher migration potential of 4-n-OP in fatty products (e.g., vegetable oils, dairy products) due to its lipophilic properties, and the stronger barrier properties of carton package when used for beverages. In conclusion, it was demonstrated that both packaging material and product composition play a decisive role in 4-n-OP contamination, with each combination carrying a unique risk profile.

The findings reveal that 4-n-NP and 4-n-OP levels vary significantly depending on the packaging type. In particular, plastic packaging shows higher mean levels of 4-n-NP and 4-n-OP in foods with high fat content, such as vegetable oils, milk, and dairy products. This can be explained by the greater solubility and migration potential of 4-n-NP and 4-n-OP in lipid-rich matrices due to their lipophilic properties. Concerns have been raised in the literature regarding the potential for 4-NP present in food packaging to migrate into the product [14], and that plastic packaging could represent a higher source of exposure to 4-NP and 4-OP [17]. Metal cans exhibited the highest mean levels of 4-n-OP, especially in the beverage group. Indeed, many studies have indicated that metal cans pose risks in terms of phenol and phthalate esters [40,41,42]. Based on the available data, carton packaging can be considered a relatively safer option in terms of 4-n-NP and 4-n-OP, while metal can (particularly for beverages) and plastic packaging (particularly for fatty foods) exhibit a more disadvantageous profile. The literature has reported that carton packaging has lower alkylphenol levels than other packaging types [27,39]. However, this assessment was based only on the target compounds measured in the study and should be evaluated within a holistic risk framework that considers not only the target analytes but also other chemical and physical risks, shelf life, and multidimensional factors such as microbial safety.

### 3.5. 4-n-NP and 4-n-OP Are Potential Sources of Contamination

Specific literature data on food contamination by the linear isomers 4-n-NP and 4-n-OP are currently limited. However, since 4-n-NP and 4-n-OP are structural isomers of 4-NP and 4-OP, contamination pathways within the food chain were evaluated based on the established sources reported for 4-NP and 4-OP. The presence of 4-NP and 4-OP in food matrices therefore indicates the potential occurrence of their linear isomers, including 4-n-NP and 4-n-OP.

Raw milk, the raw material for dairy product samples, is considered a primary source of 4-NP and 4-OP contamination. Alkylphenols can contaminate agricultural areas as a result of industrial waste being transported via air, water, and soil [43,44]. Consumption of contaminated feed and water by animals can lead to the bio-transfer of phenol derivatives into raw milk [45]. Another potential source of contamination may be the use of phenol-contaminated water in products or during cleaning [1,46]. The role of water in 4-NP and 4-OP contamination may be more pronounced, especially in dairy products with a high water content, such as ayran. Due to their high thermal stability, pasteurization and sterilization processes are not expected to significantly alter the chemical structure of these compounds. However, migration of alkylphenols such as 4-NP and 4-OP from plastic-based packaging materials is possible, particularly under conditions of high temperature, light exposure, and long storage times [17]. Guart et al. emphasized that alkylphenol migration from packaging such as polyethylene and polypropylene increases significantly depending on storage conditions and should be evaluated for consumer health [47]. Indeed, in this study, the observation that the highest levels of 4-n-NP and 4-n-OP were detected in vegetable oils sold in plastic packaging can be interpreted as a consequence of product characteristics, such as high fat content, which may facilitate alkylphenol migration under inappropriate storage conditions. Many studies have demonstrated the effect of fat content on the accumulation of alkylphenols [24,48,49]. However, another study reported that high 4-NP levels were not associated with fat content [28]. Some features of carton packages multilayer structure and aluminum foil barrier, which limit external contact, may reduce 4-n-NP and 4-n-OP migration in milk and dairy products. Nevertheless, the detection of levels similar to those in plastic packaging in some carton package samples suggests that factors such as product formulation, storage conditions, and shelf life, regardless of packaging type, may also be influential. The yeast cultures and low pH values used in the production of fermented products such as ayran and yogurt may have increased the solubility of 4-n-NP and 4-n-OP. However, the low levels of 4-n-NP and 4-n-OP in kefir, another fermented product, do not support this hypothesis. In this context, the potential influence of the microbiological properties of fermented products on alkylphenols constitutes an important area of research. Furthermore, the use of phenol-based disinfectants and other cleaning products in food production facilities has also been reported to cause contamination [50]. Therefore, a holistic food safety approach should be adopted across all stages of raw material handling, processing, storage, and packaging to prevent contamination.

The primary sources of 4-n-NP and 4-n-OP contamination in beverage samples appear to be their direct presence in product formulations and the possibility of contamination from water used in cleaning production equipment. Most of the examined beverage formulations contained a high water content. The high-temperature filling processes of RTD chilled coffee, together with the creamer and flavoring additives in the packaging materials, are considered potential sources of 4-n-OP migration. Furthermore, the fat content of milk and dairy ingredients (as in RTD chilled coffee) may also influence contamination levels. The high acidity, synthetic additives, and flavorings in energy drink and soft drink samples, along with the polymeric compounds in the inner coatings of plastic and metal can packaging, are considered to facilitate 4-NP migration [41]. In these product groups, migration risks associated with both the inner coating materials of the packaging and the additives used in production are significant, particularly in acidic beverages. Bottled water samples appear to be highly susceptible to alkylphenol migration due to environmental factors and improper storage conditions in plastic bottles, a finding consistent with results observed for plastic packaging in milk and dairy products. Iced tea and fruit juice samples may be more prone to contamination because of their additive content, highly processed nature, and packaging types designed for extended shelf life. However, the relatively low levels of alkylphenols detected in fruit juice samples may be related to the fact that most of these products are sold in carton packaging.

4-n-NP and 4-n-OP contamination in vegetable oils can result from both environmental and industrial factors, including contact with contaminated water and soil during agricultural production, pesticide residues, and migration from plastic processing equipment and packaging materials. These alkylphenols are released into the environment as degradation products of nonylphenol ethoxylates used in detergents, plasticizers, pesticide formulations, and food packaging, and can subsequently contaminate agricultural areas and water resources [50,51,52]. Vegetable oils may also be indirectly contaminated through soil and water to which the source plants are exposed. In addition, the use of plastic equipment and containers not suitable for food contact during post-harvest transportation, storage, and oil extraction can increase contamination risks. Cleaning agents applied in industrial facilities, wastewater residues in processing water systems, and solvents used during oil extraction may also contribute to the migration of 4-NP and 4-OP into oils. Plastic-based packaging, in particular, represents a critical contamination source, and appropriate transportation and storage conditions are essential to minimizing this risk. Overall, alkylphenol contamination in vegetable oils is not confined to agricultural production but represents a multi-source problem spanning the entire production chain.

### 3.6. Assessment of Dietary Exposure

The study found relatively low levels of 4-n-NP and 4-n-OP in food groups other than vegetable oils. However, exposure to these compounds through regular food consumption may exert cumulative effects over the long term, and potential endocrine-disrupting effects can occur even at low doses. Therefore, further assessment of this exposure and its associated health risks is warranted.

The average daily total phenols (4-n-NP + 4-n-OP) exposure levels resulting from the consumption of ayran, yogurt, kefir, children’s flavoured milk, and UHT milk ranged from 0.02 ± 0.03 to 0.20 ± 0.18 µg/day, with the lowest and highest values detected in kefir and children’s flavoured milk, and ayran products, respectively. Beverages were ranked by average daily total phenols exposure levels as follows: RTD chilled coffee > energy drink > soft drink > bottled water > iced tea > fruit juice. The average daily total phenols exposure levels resulting from the consumption of sunflower oil, corn oil, and olive oil were similar (Table 2).

Dietary exposure is directly related to both the contaminant concentration in a product and the amount consumed. The determining factor for dietary exposure from milk and dairy products is the concentration of 4-n-NP and 4-n-OP detected in the product. The high exposure level from ayran, despite similar consumption amounts, exemplifies this relationship. Although yogurt consumption was relatively low compared to other dairy products, the high total phenols concentrations in yogurt led to increased exposure. In the case of children’s flavoured milk, the lower body weight of children renders the same exposure dose more critical. It should also be noted that yogurt and UHT milk may be consumed more frequently and in higher quantities than ayran, which may influence the overall exposure ranking.

In the beverage group, the levels of 4-n-NP, 4-n-OP, and total phenols, as well as the consumption volume in RTD chilled coffee samples, were higher than in other beverages. In contrast, energy drinks and water, despite having similar total phenols levels, showed differences in their exposure rankings. Likewise, soft drink samples had lower total phenols levels than bottled water but were ranked higher in terms of exposure. This was primarily determined by the consumption volume of the products. Indeed, the average consumption volume of energy drinks and soft drinks was approximately 50% higher than that of bottled water (Table S2). However, given that water is an essential beverage in daily life, total 4-n-NP, 4-n-OP, and total phenols exposure from bottled water consumption may still be higher in absolute terms. Therefore, not only contamination levels but also consumption frequency and total intake volume play a critical role in assessing exposure and associated health risks.

Within the vegetable oils group, sunflower oil, which has the highest consumption, showed the lowest total phenols exposure levels, whereas olive oil, despite the lowest consumption level, showed the highest exposure. This indicates that contamination level, rather than consumption amount, is the primary determinant of exposure in this group.

Assuming a scenario in which an individual may consume these foods together within a single day, the mean levels of 4-n-NP and 4-n-OP were calculated for each food group. Accordingly, the mean daily exposure levels associated with the combined consumption of three different food groups were calculated as 0.02 ± 0.03, 0.05 ± 0.05, and 0.06 ± 0.07 µg/day for 4-n-NP, 4-n-OP, and total phenols, respectively. In the scenario where the products with the maximum 4-n-NP and 4-n-OP levels in each product group were consumed together, the exposure levels were determined as 0.52 µg/day (yogurt + energy drink + olive oil) and 3.32 µg/day (ayran + RTD chilled coffee + sunflower oil), respectively.

Toxicological studies on 4-n-NP and 4-n-OP are limited. No tolerable daily nonylphenol intake (TDI) values have been established for 4-n-NP and 4-NP. The Danish Institute of Food Safety and Toxicology proposed a tolerable daily nonylphenol intake (TDI) of 5 μg/kg bw/day for humans. Applying a safety factor of 1:100 (human:rat) resulted in a rat TDI of 0.5 mg/kg bw/day [53]. For a 70 kg adult, the human TDI corresponds to 350 μg/day. Therefore, the exposure levels determined for 4-n-NP in this study were considerably lower than this reference value. Li et al. reported exposure levels of 1.41 µg/day from the consumption of 2 L of water per day. In this study, exposure from bottled water consumption was calculated as 0.80 and 1.60 µg/day for 1 and 2 L, respectively, which is higher than previously reported values [32]. In another study conducted in Germany, total daily 4-NP exposure from foods including fruits, vegetables, milk and dairy products, coffee, and fruit juice was reported as 7.5 µg/day; however, no exposure levels were provided by food group [33]. No TDI value has been established for 4-n-OP, and no studies reporting dietary exposure levels of 4-n-OP and 4-OP were identified in the literature.

## 4. Limitations

This study has several limitations that should be considered when interpreting the results. The product set (n = 158) was selected based on availability and brand recognition rather than a probability-based national sampling design. Therefore, the findings should not be interpreted as nationally representative estimates for all products sold in Türkiye. Contamination in these products is likely multifactorial, reflecting the combined influence of food matrix characteristics, processing conditions, packaging type, and possible migration during storage. Therefore, the subgroup comparisons presented here should be interpreted cautiously, particularly where sample numbers were limited or unbalanced. The exposure assessment was calculated using the consumption data reported in Supplementary Tables S1–S3 and reflects scenario-based intake assumptions. Consumption amounts can vary across individuals as well as cultural and geographical contexts, thereby affecting estimated exposure. In addition, dietary sources containing 4-n-NP and 4-n-OP that were not included in this study, as well as non-dietary sources of exposure, were not considered.

## 5. Conclusions

This study comprehensively evaluates the levels of 4-n-NP and 4-n-OP in milk and dairy products, beverages, and vegetable oils analyzed by GC-MS/MS, with respect to product type, packaging material, and potential contamination sources. The findings revealed that contamination levels within the examined food groups showed statistically significant differences depending on product type and packaging type. Fermented products such as ayran and yogurt exhibited higher contamination profiles, particularly for 4-n-OP, while kefir and infant milk showed the lowest values for 4-n-NP and 4-n-OP. In the beverage group, RTD chilled coffee stood out with its high levels of 4-n-OP, while energy drinks showed the highest averages for 4-n-NP. Among vegetable oils, olive oil had the highest contamination levels for all target compounds, while sunflower oil had the lowest. Regarding packaging types, plastic packaging presented a higher risk profile in terms of 4-n-NP and 4-n-OP levels, particularly in fatty food groups (vegetable oils), while metal can package stood out in terms of 4-n-OP levels in beverages. Carton packaging, on the other hand, showed generally lower contamination levels. These findings suggest that the interaction between packaging material and food composition is as decisive as the contamination levels themselves. Nutrition-based exposure assessments revealed that consumption amounts are as important as contamination levels. Products with both high levels of contamination and high consumption, such as RTD chilled coffee, energy drinks, and ayran, raise concerns regarding long-term exposure and associated health risks. Olive oil, despite its lower consumption compared to other oils, was found to be risky due to its high contamination levels. Long-term, low-dose exposure to such compounds can have serious consequences, especially for children.

Overall, the data obtained in this study demonstrate that foodborne 4-n-NP and 4-n-OP exposure is a multifactorial process and that holistic strategies for risk reduction are necessary. In this context, measures to prevent contamination should be implemented at every stage of the raw material supply chain—from packaging selection to production processes, storage, and distribution—and consumer awareness campaigns should be conducted. Furthermore, establishing regular monitoring programs, especially for high-risk products, is crucial for protecting public health.

## Figures and Tables

**Table 1:** Optimized MRM transitions and collision energies for the analysis of 4-n-NP and 4-n-OP using GC-MSMS.

Compound	Retention Time (min)	Ion Polarity	Precursor Ion (m/z)	Product Ion-1 (m/z) (Quantifier)	Product Ion-2 (m/z) (Qualifier)	Dwell Time (ms)	Collision Energy (eV)
4-n-OP	10.61	Positive	206	121	135	50	10
4-n-NP	11.33	Positive	219	133	147	50	10
Phenol-d6 *	5.78	Positive	100	84	66	50	10

* Internal Standarf (IS), 4-n-NP: 4-n-Nonylphenol, 4-n-OP: 4-n-Octylphenol.

**Table 2:** Daily exposure levels of 4-n-NP, 4-n-OP, and total phenols (4-n-NP + 4-n-OP) through consumption of milk and dairy products, beverages, and vegetable oils (µg/day).

Products	4-n-NP			4-n-OP			Total Phenols		
	Mean ± SD	Min.	Max.	Mean ± SD	Min.	Max.	Mean ± SD	Min.	Max.
Milk and dairy products									
Ayran	0.02 ± 0.05	0.00	0.09	0.18 ± 0.21	0.00	0.38	0.20 ± 0.18	0.00	0.38
Yogurt	0.03 ± 0.08	0.00	0.27	0.07 ± 0.07	0.00	0.26	0.10 ± 0.11	0.00	0.36
Kefir	0.00 ± 0.00	0.00	0.00	0.02 ± 0.04	0.00	0.10	0.02 ± 0.04	0.00	0.01
Children’s flavoured milk	0.02 ± 0.03	0.00	0.08	0.00 ± 0.00	0.00	0.00	0.02 ± 0.03	0.00	0.08
UHT milk	0.04 ± 0.06	0.00	0.18	0.02 ± 0.04	0.00	0.10	0.05 ± 0.06	0.00	0.18
Beverages									
Soft drink	0.04 ± 0.05	0.00	0.21	0.06 ± 0.05	0.00	0.15	0.10 ± 0.06	0.00	0.22
Iced tea	0.06 ± 0.04	0.00	0.09	0.00 ± 0.00	0.00	0.00	0.06 ± 0.02	0.00	0.09
Energy drink	0.08 ± 0.11	0.00	0.24	0.04 ± 0.07	0.00	0.19	0.12 ± 0.12	0.00	0.34
Fruit juice	0.05 ± 0.05	0.00	0.13	0.00 ± 0.00	0.00	0.00	0.05 ± 0.05	0.00	0.13
RTD chilled coffee	0.02 ± 0.04	0.00	0.10	0.36 ± 1.03	0.00	2.91	0.38 ± 1.02	0.00	2.91
Bottled water	0.03 ± 0.03	0.00	0.07	0.04 ± 0.12	0.00	0.34	0.07 ± 0.11	0.00	0.34
Vegetable oils									
Sunflower oil	<0.00 ± 0.00	0.00	<0.00	0.01 ± 0.01	0.00	0.03	0.01 ± 0.01	0.00	0.04
Corn oil	<0.00 ± 0.00	0.00	<0.00	0.01 ± 0.01	0.00	0.02	0.01 ± 0.01	<0.00	0.02
Olive oil	<0.00 ± 0.00	0.00	0.01	0.01 ± 0.01	0.00	0.02	0.01 ± 0.01	<0.00	0.02

Daily dietary exposure levels were calculated according to Equation 1, based on the consumption data provided in Supplementary Tables S1–S3.

## Data Availability

The original contributions presented in this study are included in the article/Supplementary Material. Further inquiries can be directed to the corresponding author.

## Supplementary Materials

The following supporting information can be downloaded at: https://www.mdpi.com/article/10.3390/foods15061063/s1, Table S1. Some information about the dairy product samples; Table S2. Some information about the beverage samples; Table S3. Some information about the vegetable oil samples; Table S4. 4-n-NP and 4-n-OP levels in milk and dairy products (µg/kg); Table S5. 4-n-NP and 4-n-OP levels in beverages (µg/L); Table S6. 4-n-NP and 4-n-OP levels in vegetable oils (µg/kg); Table S7. 4-n-NP and 4-n-OP levels according to packaging types (µg/kg).
