# Supplementary material for: Alkylphenols (4-n-Nonylphenol and 4-n-Octylphenol) in Milk and Dairy Products, Beverages, and Vegetable Oils: Occurrence and Dietary Exposure in Türkiye
Source: Foods. 2026 Mar 18;15(6):1063. doi: 10.3390/foods15061063 (PMC13025704; doi:10.3390/foods15061063)
Supplement: Supplementary file 1 [file foods-15-01063-s001.zip › foods-4195385-supplementary.pdf]

Alkylphenols (4-n-Nonylphenol and 4-n-Octylphenol) in Milk and Dairy Products, Beverages, and Vegetable Oils: Occurrence and Dietary Exposure in Türkiye

Oltan Canlı [1], Barış Güzel [1] and Burhan Basaran [2,*]

[1] Climate Studies and Water Management Research Group, Climate and Life Vice Presidency, TUBITAK Marmara Research Center, 41470 Kocaeli, Türkiye; oltan.canli@tubitak.gov.tr (O.C.); guzelbaris08@gmail.com (B.G.)

[2] Department of Nutrition and Dietetics, Faculty of Health Sciences, Recep Tayyip Erdogan University, 53100 Rize, Türkiye

* Correspondence: burhan.basaran@erdogan.edu.tr

Orcid Numbers of All Authors:

Barış Güzel: 0000-0002-6709-2339

Oltan Canlı: 0000-0002-2652-844X

Burhan Başaran: 0000-0001-6506-6113

Table S1. Some information about the dairy product samples.

Sample No	Packaging type	Product features	Packaging volume/ Consumption amount
Sample 1	Plastic	Buttermilk	170 mL
Sample 2	Plastic	Buttermilk	200 mL
Sample 3	Plastic	Buttermilk	200 mL
Sample 4	Plastic	Buttermilk	200 mL
Sample 5	Plastic	Yogurt	200 g
Sample 6	Plastic	Yogurt	200 g
Sample 7	Plastic	Yogurt	200 g
Sample 8	Plastic	Yogurt	200 g
Sample 9	Plastic	Fruit yogurt	67 g
Sample 10	Plastic	Strawberry yogurt	65 g
Sample 11	Plastic	Strawberry yogurt	90 g
Sample 12	Plastic	Strawberry yogurt	125 g
Sample 13	Plastic	Strawberry probiotic yogurt	100 g
Sample 14	Plastic	Fruit yogurt	90 g
Sample 15	Plastic	Probiotic yogurt	100 g
Sample 16	Plastic	Fruit yogurt	125 g
Sample 17	Plastic	Strawberry kefir	330 mL
Sample 18	Plastic	Kefir	330 mL
Sample 19	Plastic	Kefir	330 mL
Sample 20	Plastic	Strawberry kefir	200 mL
Sample 21	Plastic	Kefir	250 mL
Sample 22	Plastic	Kefir	290 mL
Sample 23	Carton package	Children's milk (Strawberry)	180 mL
Sample 24	Carton package	Children's milk (Strawberry)	180 mL
Sample 25	Carton package	Children's milk (Strawberry)	200 mL
Sample 26	Carton package	Children's milk	330 mL
Sample 27	Carton package	Children's milk (Banana)	180 mL
Sample 28	Carton package	Children's milk (Banana)	200 mL
Sample 29	Carton package	Children's milk (Banana)	200 mL
Sample 30	Carton package	Children's milk (Cocoa)	180 mL
Sample 31	Carton package	Children's milk (Banana)	180 mL
Sample 32	Carton package	Children's milk (Banana)	200 mL
Sample 33	Carton package	Children's milk (Banana)	180 mL
Sample 34	Carton package	Children's milk (Cocoa)	200 mL
Sample 35	Carton package	Children's milk (Cocoa)	180 mL
Sample 36	Carton package	Children's milk (Cocoa)	180 mL
Sample 37	Carton package	Children's milk (Cocoa)	200 mL
Sample 38	Carton package	Children's milk (Cocoa)	180 mL
Sample 39	Carton package	Children's milk (Cocoa)	200 mL
Sample 40	Carton package	UHT Milk	200 mL
Sample 41	Carton package	UHT Milk	250 mL
Sample 42	Carton package	UHT Milk	250 mL
Sample 43	Carton package	UHT Milk	200 mL
Sample 44	Carton package	UHT Milk	200 mL
Sample 45	Carton package	UHT Milk	200 mL
Sample 46	Carton package	UHT Milk	200 mL
Sample 47	Carton package	UHT Milk	200 mL
Sample 48	Carton package	UHT Milk	200 mL
Sample 49	Carton package	UHT Milk	200 mL
Sample 50	Carton package	UHT Milk	200 mL
Sample 51	Carton package	UHT Milk	200 mL
Sample 52	Carton package	UHT Milk	200 mL
Sample 53	Carton package	UHT Milk	200 mL
Sample 54	Carton package	UHT Milk	200 mL

Table S2. Some information about the beverage samples.

Sample No	Packaging type	Product features	Consumption amount
Sample 55	Plastic	Soft drink (blue)	330 mL
Sample 56	Plastic	Soft drink (cola)	330 mL
Sample 57	Plastic	Soft drink (flavored soda)	330 mL
Sample 58	Plastic	Soft drink (orange-flavored)	330 mL
Sample 59	Plastic	Soft drink (orange-flavored)	330 mL
Sample 60	Plastic	Soft drink (orange-flavored)	330 mL
Sample 61	Plastic	Soft drink (orange-flavored)	330 mL
Sample 62	Plastic	Soft drink (cola)	330 mL
Sample 63	Plastic	Soft drink (flavored soda)	330 mL
Sample 64	Plastic	Soft drink (cola)	330 mL
Sample 65	Plastic	Soft drink (cola)	330 mL
Sample 66	Plastic	Soft drink (lemonade)	200 mL
Sample 67	Plastic	Soft drink (lemonade)	200 mL
Sample 68	Plastic	Soft drink (lemonade)	200 mL
Sample 69	Plastic	Soft drink (lemonade)	200 mL
Sample 70	Metal can	Soft drink (cola)	330 mL
Sample 71	Metal can	Soft drink (orange-flavored)	330 mL
Sample 72	Metal can	Soft drink (cola)	330 mL
Sample 73	Metal can	Soft drink (cola)	330 mL
Sample 74	Metal can	Soft drink (orange-flavored)	330 mL
Sample 75	Metal can	Soft drink (flavored soda)	330 mL
Sample 76	Metal can	Soft drink (cola)	330 mL
Sample 77	Metal can	Soft drink (cola)	330 mL
Sample 78	Metal can	Soft drink (flavored soda)	330 mL
Sample 79	Metal can	Soft drink (flavored soda)	330 mL
Sample 80	Metal can	Soft drink (flavored soda)	330 mL
Sample 81	Metal can	Soft drink (cola)	330 mL
Sample 82	Metal can	Soft drink (orange-flavored)	330 mL
Sample 83	Plastic	Fruit juice (Traditional turnip juice)	200 mL
Sample 84	Plastic	Fruit juice (Traditional turnip juice)	200 mL
Sample 85	Plastic	Fruit juice (Traditional turnip juice)	200 mL
Sample 86	Plastic	Fruit juice (Traditional turnip juice)	200 mL
Sample 87	Plastic	Fruit juice (cherry)	200 mL
Sample 88	Plastic	Fruit juice (orange)	200 mL
Sample 89	Carton package	Fruit juice (mixed)	200 mL
Sample 90	Carton package	Fruit juice (mixed)	200 mL
Sample 91	Carton package	Fruit juice (mixed)	200 mL
Sample 92	Carton package	Fruit juice (cherry)	200 mL
Sample 93	Carton package	Fruit juice (cherry)	200 mL
Sample 94	Carton package	Fruit juice (apricot)	200 mL
Sample 95	Carton package	Fruit juice (mixed)	200 mL
Sample 96	Carton package	Fruit juice (mixed)	200 mL
Sample 97	Carton package	Fruit juice (peach)	200 mL
Sample 98	Carton package	Fruit juice (cherry)	200 mL
Sample 99	Carton package	Fruit juice (apricot)	200 mL
Sample 100	Carton package	Fruit juice (apricot)	200 mL
Sample 101	Carton package	Fruit juice (orange)	200 mL
Sample 102	Plastic	Energy drink	330 mL
Sample 103	Metal can	Energy drink	330 mL
Sample 104	Metal can	Energy drink	330 mL
Sample 105	Metal can	Energy drink	330 mL
Sample 106	Metal can	Energy drink	330 mL
Sample 107	Metal can	Energy drink	330 mL
Sample 108	Metal can	Energy drink	330 mL
Sample 109	Metal can	Energy drink (vitamin C)	330 mL
Sample 110	Metal can	Energy drink (mutlivitamin)	330 mL
Sample 111	Plastic	Bottled water	200 mL
Sample 112	Plastic	Bottled water	200 mL
Sample 113	Plastic	Bottled water	200 mL
Sample 114	Plastic	Bottled water	200 mL

Sample 115	Plastic	Bottled water	200 mL
Sample 116	Plastic	Bottled water	200 mL
Sample 117	Plastic	Bottled water	200 mL
Sample 118	Plastic	Bottled water	200 mL
Sample 119	Metal can	RTD chilled coffee (latte)	250 mL
Sample 120	Metal can	RTD chilled coffee (latte)	250 mL
Sample 121	Metal can	RTD chilled coffee (latte)	250 mL
Sample 122	Metal can	RTD chilled coffee (latte)	250 mL
Sample 123	Metal can	RTD chilled coffee (cappucino)	250 mL
Sample 124	Metal can	RTD chilled coffee (cappucino)	250 mL
Sample 125	Metal can	RTD chilled coffee (mocca)	250 mL
Sample 126	Metal can	RTD chilled coffee (latte)	250 mL
Sample 127	Metal can	Iced tea	330 mL
Sample 128	Metal can	Iced tea	330 mL
Sample 129	Metal can	Iced tea	330 mL
Sample 130	Metal can	Iced tea	330 mL
Sample 131	Metal can	Iced tea	330 mL
Sample 132	Metal can	Iced tea	330 mL
Sample 133	Metal can	Iced tea	330 mL

Table S3. Some information about the vegetable oil samples.

Sample No	Packaging type	Product features	Consumption amount
Sample 134	Plastic	Sunflower oil	16.6 mL
Sample 135	Plastic	Sunflower oil	16.6 mL
Sample 136	Plastic	Sunflower oil	16.6 mL
Sample 137	Plastic	Sunflower oil	16.6 mL
Sample 138	Plastic	Sunflower oil	16.6 mL
Sample 139	Plastic	Sunflower oil	16.6 mL
Sample 140	Plastic	Sunflower oil	16.6 mL
Sample 141	Plastic	Sunflower oil	16.6 mL
Sample 142	Plastic	Sunflower oil	16.6 mL
Sample 143	Plastic	Corn oil	10 mL
Sample 144	Plastic	Corn oil	10 mL
Sample 145	Plastic	Corn oil	10 mL
Sample 146	Plastic	Corn oil	10 mL
Sample 147	Plastic	Corn oil	10 mL
Sample 148	Plastic	Corn oil	10 mL
Sample 149	Plastic	Corn oil	10 mL
Sample 150	Plastic	Olive oil	5.2 mL
Sample 151	Plastic	Olive oil	5.2 mL
Sample 152	Plastic	Olive oil	5.2 mL
Sample 153	Plastic	Olive oil	5.2 mL
Sample 154	Plastic	Olive oil	5.2 mL
Sample 155	Plastic	Olive oil	5.2 mL
Sample 156	Plastic	Olive oil	5.2 mL
Sample 157	Plastic	Olive oil	5.2 mL
Sample 158	Plastic	Olive oil	5.2 mL

Table S4. 4-n-NP and 4-n-OP levels in milk and dairy products (µg/kg).

Products	4-n-NP			4-n-OP		
	Median	Min.	Max.	Median	Min.	Max.
Ayran	0.00[b]	<LOD	0.56	0.82[a]	<LOD	1.90
Yogurt	0.00[a]	<LOD	1.33	0.00[b]	<LOD	1.91
Kefir	0.00[c]	<LOD	<LOQ	0.00[c]	<LOD	0.41
Children's flavoured milk	0.00[b]	<LOD	0.40	0.00[c]	<LOD	<LOQ
UHT milk	0.00[ab]	<LOD	0.88	0.00[c]	<LOD	0.51

Different letters within the same group indicate statistically significant differences ($p<0.05$).

Table S5. 4-n-NP and 4-n-OP levels in beverages (µg/L).

Products	4-n-NP			4-n-OP		
	**Median**	**Min.**	**Max.**	**Median**	**Min.**	**Max.**
Soft drink	0.04[b]	<LOD	0.63	0.21[a]	<LOD	0.46
Iced tea	0.24[a]	<LOD	0.28	0.00[b]	<LOD	<LOQ
Energy drink	0.09[b]	<LOD	0.71	0.00[b]	<LOD	0.56
Fruit juice	0.19[a]	<LOD	0.66	0.00[b]	<LOD	<LOQ
RTD chilled coffee	0.00[c]	<LOD	0.38	0.00[a]	<LOD	11.6
Bottled water	0.14[b]	<LOD	0.37	0.00[a]	<LOD	1.70

Different letters within the same group indicate statistically significant differences ($p<0.05$).

Table S6. 4-n-NP and 4-n-OP levels in vegetable oils (μg/kg).

Products	4-n-NP			4-n-OP		
	**Median**	**Min.**	**Max.**	**Median**	**Min.**	**Max.**
Sunflower oil	0.00[c]	<LOD	0.30	0.12[b]	<LOD	1.89
Corn oil	0.26[b]	<LOD	0.41	1.21[a]	<LOD	1.52
Olive oil	0.48[a]	0.12	0.96	0.69[a]	<LOD	3.37

Different letters within the same group indicate statistically significant differences ($p<0.05$).

Table S7. 4-n-NP and 4-n-OP levels according to packaging types (μg/kg).

Products	Packaging types	4-n-NP			4-n-OP			4-n-NP + 4-n-OP		
		**Median**	**Min.**	**Max.**	**Median**	**Min.**	**Max.**	**Median**	**Min.**	**Max.**
Beverages	Metal can	0.09^{a}	<LOD	0.71	0.00^{a}	<LOD	11.6	0.27^{a}	<LOD	11.6
	Plastic	0.16^{a,2}	<LOD	0.66	0.00^{b,3}	<LOD	1.70	0.29^{a,2}	<LOD	1.70
	Carton package	0.12^{a,1}	<LOD	0.65	0.00^{b,2}	<LOD	<LOQ	0.12^{b,1}	<LOD	0.65
Milk and dairy products	Plastic	0.00^{a,2}	<LOD	1.33	0.44^{a,2}	<LOD	1.91	0.53^{a,2}	<LOD	1.91
	Carton package	0.00^{b,1}	<LOD	0.88	0.00^{b,1}	<LOD	0.51	0.00^{b,1}	<LOD	0.88
Vegetable oils	Plastic	0.26^{1}	<LOD	0.96	0.58^{1}	<LOD	3.37	0.81^{1}	<LOD	3.78

Different letters or numbers within the same group indicate statistically significant differences ($p<0.05$).
